# Tuberculous Lymphadenitis Presenting as Chest Wall Cold Abscess: A Case Report

**DOI:** 10.7759/cureus.107333

**Published:** 2026-04-19

**Authors:** Garima Chandra

**Affiliations:** 1 Internal Medicine, Yenepoya Medical College, Mangalore, IND

**Keywords:** anti-tubercular therapy, chest wall abscess, cold abscess, extrapulmonary tuberculosis, granulomatous inflammation, rib osteolysis, tuberculosis, tuberculous lymphadenitis

## Abstract

Tuberculosis (TB) is a significant global health challenge, with chest wall involvement representing a rare extrapulmonary manifestation. Tuberculous cold abscess of the chest wall is particularly infrequent.

This case describes a 59-year-old female with chronic back pain and a non-tender swelling on the anterolateral chest wall. Advanced imaging revealed enlarged, conglomerate subcarinal lymphadenopathy and a cold abscess associated with osteolytic destruction of the ninth rib, without pulmonary involvement. Microbiological evaluation confirmed the presence of *Mycobacterium tuberculosis*, and cytological examination demonstrated granulomatous inflammation with caseous necrosis and Langhans giant cells, supportive of TB. The patient was treated with a weight-based, first-line antitubercular therapy regimen for 11 months. Complete resolution of the swelling was achieved by the fifth month, and the patient remained asymptomatic, with no recurrence at the final follow-up.

This case posed a significant diagnostic challenge, as chest wall TB can mimic pyogenic infections or primary chest wall malignancies, making imaging and microbiological testing essential for accurate diagnosis and treatment.

## Introduction

Tuberculosis (TB) remains a major public health problem, particularly in developing nations, and may present with multisystem involvement [1,2]. While pulmonary involvement is most common, extrapulmonary TB accounts for 20%-30% of all active cases [3]. Within extrapulmonary cases, musculoskeletal TB is estimated to account for approximately 10% of cases in some populations, though this proportion may vary geographically [4]. Involvement of the chest wall is rare and often presents a diagnostic challenge, as it may mimic pyogenic infections or malignancies [1,5,6].

The pathogenesis of chest wall TB typically involves three mechanisms: hematogenous dissemination, contiguous extension from pleuropulmonary sites, or direct extension from lymphadenitis [1,7]. The latter is most commonly implicated in the development of subcutaneous cold abscesses, wherein infection of the mediastinal or intercostal lymph nodes tracks through the wall to form a visible, non-tender swelling [1,2].

In this case, we describe an unusual presentation of a cold abscess overlying the right ninth rib, secondary to tuberculous lymphadenitis of the subcarinal lymph nodes, illustrating an uncommon route of extension and emphasizing the need for advanced imaging and microbiological confirmation in establishing the diagnosis.

## Case presentation

A 59-year-old female with no prior comorbidities presented for evaluation of chronic upper back pain and swelling over the right anterolateral chest wall. The patient reported persistent back pain for one year, localized to the upper back (T4-T9 vertebral level). Over time, the patient noticed the development of a swelling over the right side of the chest wall, which gradually increased in size. She denied constitutional symptoms such as weight loss or evening rise of temperature. There was no history of cough, breathlessness, chest pain, hemoptysis, or prior TB exposure.

On examination, the patient was moderately built and nourished, weighing 67 kg and measuring 150 cm, with a body mass index of 29.8 kg/m². She was afebrile and hemodynamically stable. Local examination revealed a non-tender swelling measuring 3 × 2 cm over the right anterolateral chest wall along the anterior axillary line, with no local rise in temperature. Respiratory system examination was unremarkable. No other abnormalities were noted on systemic examination.

Laboratory investigations demonstrated persistently elevated erythrocyte sedimentation rate (ESR) and thrombocytosis over the preceding two years. White blood cell counts and hemoglobin levels were within normal limits. The patient had no prior history of immunosuppressive therapy, including long-term corticosteroid use, chemotherapy, or biological agents, and was not on any chronic medications. The patient was evaluated for immunocompromised states with HIV serology and fasting blood glucose/HbA1c testing, all of which were within normal limits.

**Figure 1:**
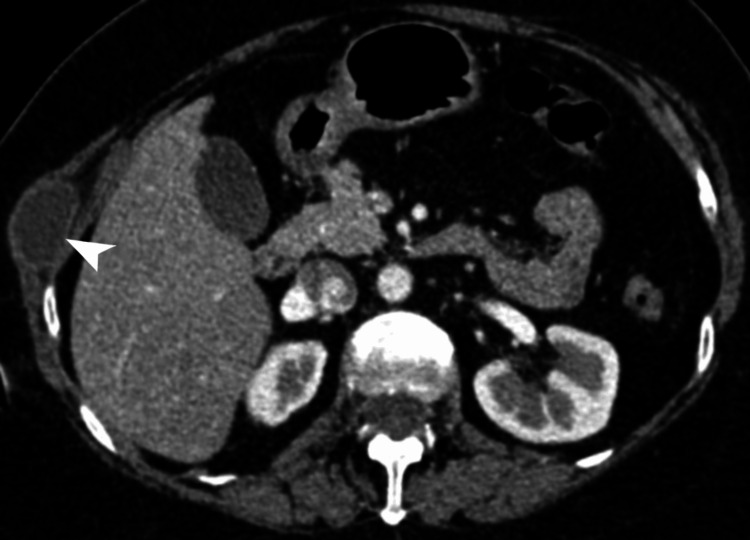
Right chest wall cystic lesion. Axial contrast-enhanced CT of the abdomen, in the soft tissue window, demonstrates a well-defined cystic lesion (white arrowhead) with peripheral enhancement, measuring 3.3 × 2.4 cm, in the right chest wall. CT: computed tomography

**Figure 2:**
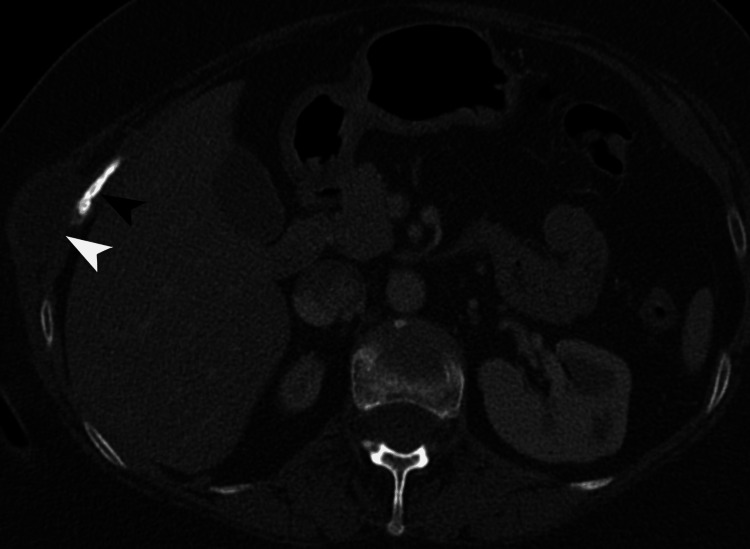
Right chest wall cystic lesion with rib erosion. Axial contrast-enhanced CT of the abdomen, in the bone window, demonstrates a well-defined cystic lesion (white arrowhead) with peripheral enhancement, measuring 3.3 × 2.4 cm, in the right chest wall, causing erosion of the ninth rib anterolaterally (black arrowhead). CT: computed tomography

**Figure 3:**
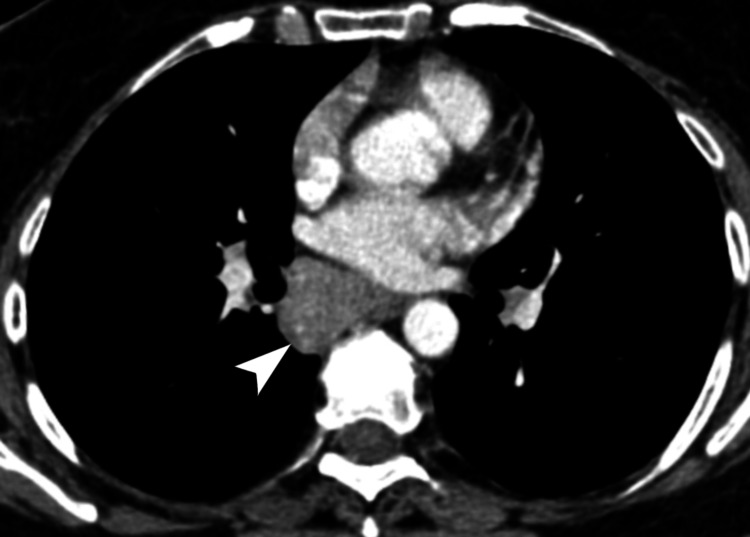
Subcarinal nodal mass with esophageal and airway encasement. Axial CT of the chest, in the soft tissue window, demonstrates a homogeneously enhancing nodal mass in the subcarinal region (white arrowhead), measuring 4.7 × 2.5 × 5.8 cm (transverse × AP × craniocaudal), partially encasing the thoracic esophagus and both the right and left main bronchi. CT: computed tomography

**Figure 4:**
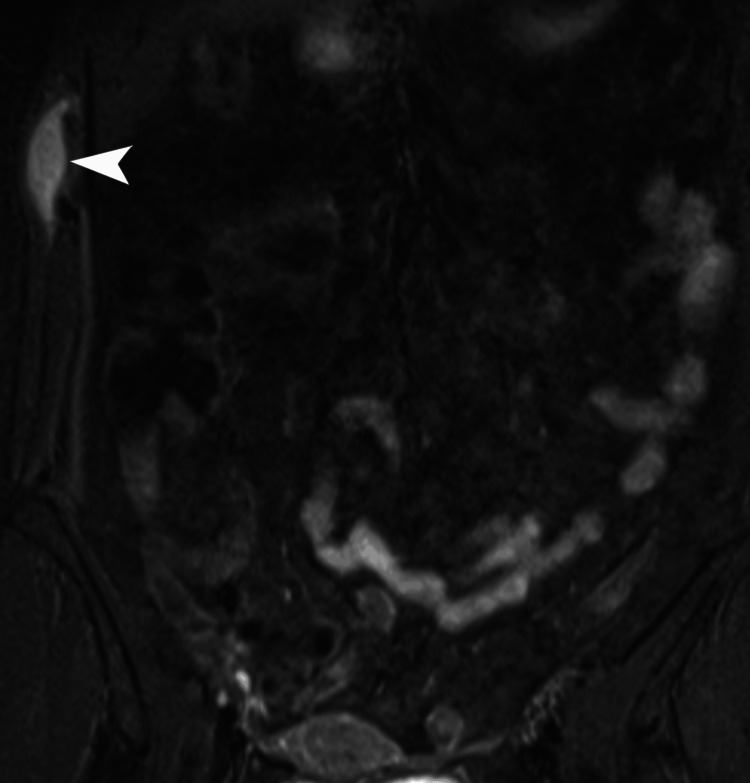
MRI of the right chest wall cystic lesion. Coronal MRI of the chest and abdomen demonstrates a T2-hyperintense lesion with fluid signal intensity along the right lateral abdominal wall (white arrowhead). MRI: magnetic resonance imaging

**Figure 5:**
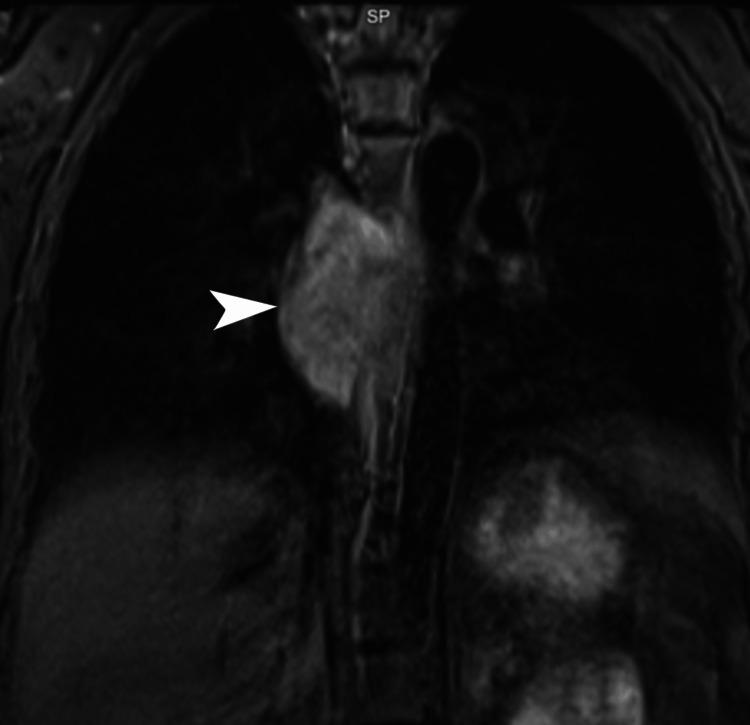
MRI of the subcarinal nodal mass. Coronal T2-weighted MRI images of the chest demonstrate a T2-hyperintense nodal mass in the subcarinal region with bilateral extension (white arrowhead). The lesion is seen producing extrinsic compression of the thoracic esophagus and extending superiorly toward the aortopulmonary region. MRI: magnetic resonance imaging

**Figure 6:**
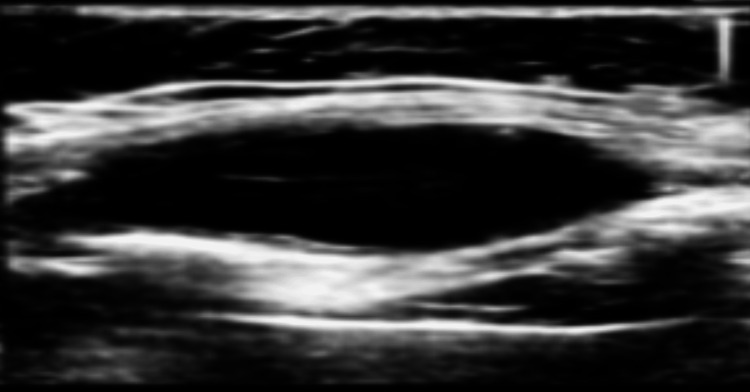
Ultrasound of the chest wall abscess. Ultrasound images of the right chest wall demonstrate a well-defined hypoechoic collection with a thick wall along the lower rib, measuring approximately 4.0 × 1.4 × 2.8 cm (estimated volume ~8.8 mL). The adjacent rib shows cortical irregularity, suggestive of underlying osseous involvement.

A chest radiograph showed no pulmonary parenchymal abnormalities or evidence of active or healed pulmonary TB. Further evaluation was performed using contrast-enhanced computed tomography (CT), magnetic resonance imaging (MRI), and ultrasonography. Imaging revealed a well-defined cystic lesion in the right chest wall with associated rib erosion (Figures 1-2). Imaging also demonstrated enlarged subcarinal lymph nodes forming a conglomerate nodal mass (Figure 3). MRI further characterized these findings, showing a T2-hyperintense lesion along the right lateral abdominal wall (Figure 4) and a T2-hyperintense subcarinal nodal mass with bilateral extension and compression of adjacent structures (Figure 5). Ultrasound confirmed a well-defined hypoechoic collection with features suggestive of osseous involvement (Figure 6).

Ultrasound-guided fine-needle aspiration and core biopsy of the abscess were performed for histopathological and microbiological evaluation, yielding 35 mL of purulent material.

Microbiological evaluation included GeneXpert *Mycobacterium tuberculosis*/Rifampicin Ultra (MTB/RIF Ultra) testing, liquid culture using the Mycobacterium Growth Indicator Tube (MGIT) system, fungal cultures, and aerobic and anaerobic cultures. The GeneXpert assay detected low levels of *M. tuberculosis*, with no rifampicin resistance. Aerobic, anaerobic, and fungal cultures were negative. MGIT culture subsequently demonstrated growth of *M. tuberculosis* complex. Drug susceptibility testing (DST), performed using the MGIT-14 drug panel, revealed sensitivity to all first-line anti-tubercular drugs, including rifampicin, isoniazid, pyrazinamide, and ethambutol, with no evidence of drug resistance. Cytological examination of the aspirated material demonstrated caseous necrosis with epithelioid cells and Langhans giant cells, supporting the diagnosis of TB.

Standard first-line anti-tubercular therapy (ATT) was initiated the day following biopsy with weight-based dosing of rifampicin, isoniazid, pyrazinamide, and ethambutol. DST was performed using GeneXpert MTB/RIF Ultra, which demonstrated no rifampicin resistance, following which standard first-line ATT was initiated. Prophylactic measures included baseline and periodic monitoring of liver function tests, complete blood counts, and visual acuity assessment; pyridoxine supplementation was administered to prevent isoniazid-induced neuropathy, and the patient was counseled regarding recognition of hepatotoxicity, visual disturbances, and other potential adverse effects.

Follow-up and outcome

**Figure 7:**
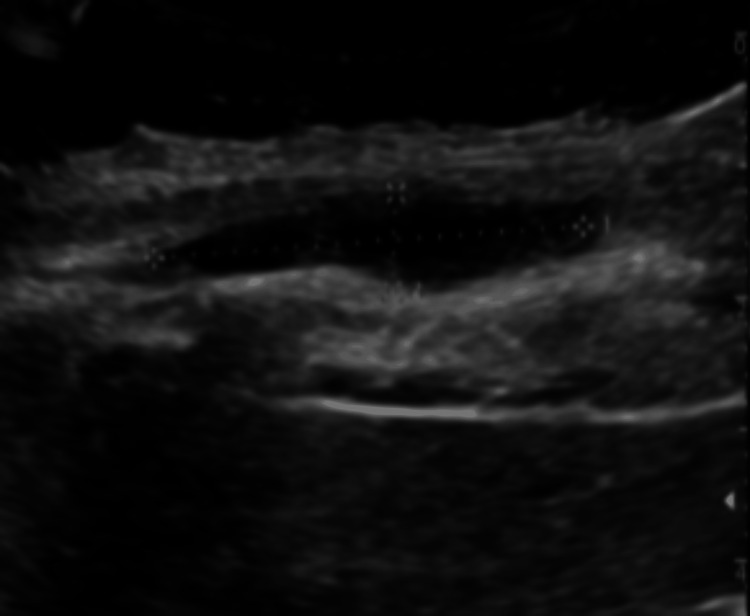
Decreasing chest wall lesion at one-month follow-up. Follow-up ultrasound of the right anterior chest wall at one month demonstrates a residual hypoechoic collection measuring approximately 3.3 × 0.7 × 0.7 cm (estimated volume ~1 mL). The collection is markedly reduced in size compared with the prior study.

**Figure 8:**
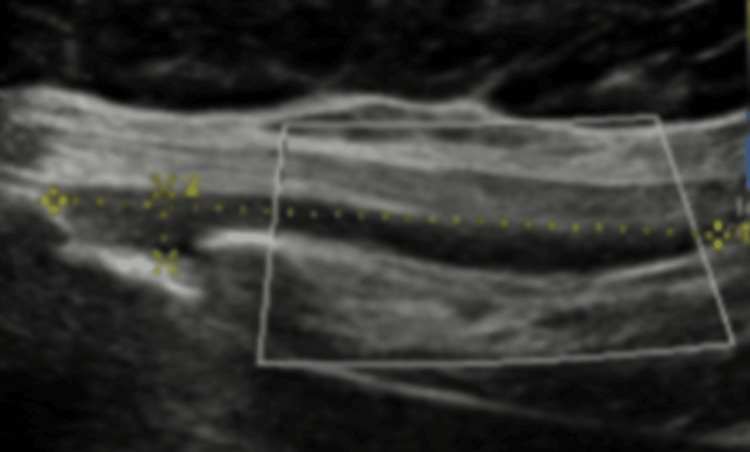
Further reduction of chest wall lesion at two-month follow-up. Follow-up ultrasound of the right anterior chest wall at two months demonstrates a small residual hypoechoic collection measuring approximately 4.1 × 0.5 × 0.5 cm (estimated volume ~0.5 mL). The collection shows a further reduction in size compared with the prior study.

**Figure 9:**
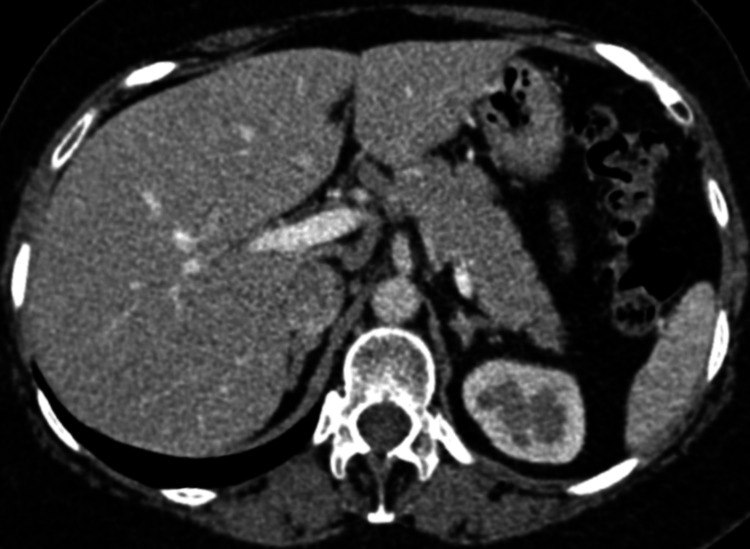
Resolution of chest wall cystic lesion with rib erosion at five-month follow-up. Axial contrast-enhanced CT image, in the soft tissue window, obtained at five-month follow-up, demonstrates complete resolution of the cystic lesion. Minimal, persistent erosion of the underlying ninth rib can be seen anterolaterally. CT: computed tomography

**Figure 10:**
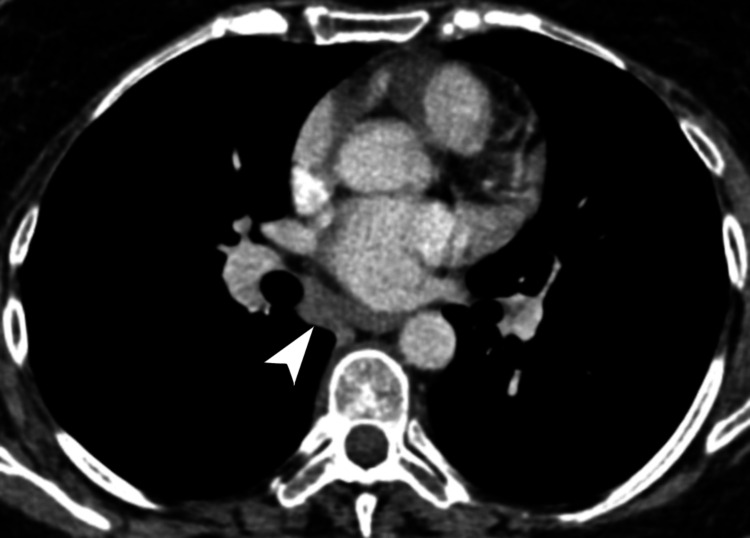
Reduction of subcarinal nodal mass at five-month follow-up. Axial CT of the chest obtained at five-month follow-up demonstrates a residual subcarinal nodal mass (white arrowhead) measuring 3.9 × 1.4 × 4.3 cm (transverse × AP × craniocaudal) with mild compression of the thoracic esophagus. CT: computed tomography

The patient demonstrated gradual clinical improvement, with progressive reduction in the size of the chest wall lesion. Follow-up ultrasound showed marked reduction in the size of the collection at one-month and two-month intervals (Figures 7-8). Follow-up CT imaging at five months demonstrated complete resolution of the chest wall lesion with residual rib erosion (Figure 9) and reduction in the size of the subcarinal nodal mass (Figure 10). Antitubercular therapy was continued for a total duration of 11 months, with complete resolution of the swelling by the fifth month of therapy. At the final follow-up, conducted six months after completion of ATT, the patient was asymptomatic, with no evidence of residual disease or recurrence on imaging.

## Discussion

Chest wall tuberculous cold abscesses are frequently difficult to identify early owing to their paucibacillary nature and relatively indolent clinical presentation [4,5]. The differential diagnosis is broad and includes pyogenic abscesses, enchondromas, and malignant tumors [1,6]. In the present case, the origin was attributed to tuberculous lymphadenitis of the subcarinal nodes, gradually extending into the adjacent chest wall to form a cold abscess.

This case illustrates a clear clinicopathological correlation in which subcarinal lymphadenitis served as the primary focus, with contiguous spread along fascial planes leading to chest wall involvement. This pattern aligns with one of the classical pathophysiological mechanisms of extrapulmonary TB, i.e., direct extension from infected lymph nodes. The presence of caseating granulomas with Langhans giant cells further supports the role of a delayed type IV hypersensitivity response, central to TB pathogenesis. Radiological evaluation was pivotal in this case, with cross-sectional imaging demonstrating a well-defined cystic lesion with rib osteolysis and associated mediastinal lymphadenopathy. CT findings of osteolysis and T2-hyperintense collections on MRI favor a cold abscess rather than an acute pyogenic process, which would exhibit more aggressive inflammatory changes. The absence of pulmonary parenchymal disease further highlights the importance of imaging in detecting occult extrapulmonary sources.

The differential diagnosis in such presentations can include not only pyogenic abscesses and malignancies but also actinomycosis, fungal infections, metastatic lesions, multiple myeloma, and primary bone tumors such as Ewing sarcoma or chondrosarcoma. While clinical features such as indolent progression, minimal systemic toxicity, and characteristic imaging findings may help guide diagnosis, definitive differentiation ultimately relies on microbiological confirmation.

Management strategies

The role of surgical intervention in chest wall TB continues to be individualized, as some patients respond well to medical therapy alone, while others may experience recurrence of the collection without adequate drainage or debridement [1,7]. Wide surgical debridement, involving the removal of all necrotic tissue and curettage of affected bone, is recommended to obliterate the residual cavity and minimize local complications [1,2]. In the present case, a conservative approach with image-guided aspiration and prolonged ATT resulted in complete resolution, supporting current evidence that select patients without extensive necrosis or sinus formation can be successfully managed non-surgically.

Anti-tubercular therapy (ATT)

Standard chemotherapy involves a four-drug regimen (isoniazid, rifampicin, pyrazinamide, and ethambutol). In patients with osteoarticular or extensive extrapulmonary involvement, ATT beyond the standard six-month regimen is considered to ensure complete resolution and reduce recurrence [1,7,8]. The extended duration of therapy (11 months) in this case aligns with literature suggesting that extrapulmonary TB often requires prolonged treatment due to poor penetration and the risk of recurrence. Studies have reported treatment durations ranging from 9 to 12 months in similar cases, with favorable outcomes when therapy is adequately administered.

## Conclusions

This case emphasizes the importance of maintaining a high degree of suspicion for TB in patients with chest wall swellings with atypical features, especially in endemic settings. Although pulmonary TB is most common, extrapulmonary forms like tuberculous lymphadenitis can present unusually, such as chest wall cold abscesses. The absence of classic symptoms may delay diagnosis, making thorough evaluation, imaging, and microbiological confirmation crucial.

Early diagnosis and initiation of anti-tubercular therapy are essential to prevent complications such as bone destruction, recurrence, or unnecessary surgical intervention. A multidisciplinary approach - integrating radiological, pathological, and microbiological findings - guides management decisions. Awareness of such rare presentations improves the timely diagnosis and treatment of extrapulmonary TB.
